# Probing microtubule polymerisation state at single kinetochores during metaphase chromosome motion

**DOI:** 10.1242/jcs.168682

**Published:** 2015-05-15

**Authors:** Jonathan W. Armond, Elina Vladimirou, Muriel Erent, Andrew D. McAinsh, Nigel J. Burroughs

**Affiliations:** 1 Warwick Systems Biology Centre and Mathematics Institute, University of Warwick, Coventry CV4 7AL, UK; 2 Mechanochemical Cell Biology Building, Division of Biomedical Cell Biology, Warwick Medical School, University of Warwick, Coventry CV4 7AL, UK

**Keywords:** K-fibres, KIF18A, MCAK, Kinetochores, Mitosis, Kinesin, EB3

## Abstract

Kinetochores regulate the dynamics of attached microtubule bundles (kinetochore-fibres, K-fibres) to generate the forces necessary for chromosome movements in mitosis. Current models suggest that poleward-moving kinetochores are attached to depolymerising K-fibres and anti-poleward-moving kinetochores to polymerising K-fibres. How the dynamics of individual microtubules within the K-fibre relate to poleward and anti-poleward movements is poorly understood. To investigate this, we developed a live-cell imaging assay combined with computational image analysis that allows eGFP-tagged EB3 (also known as MAPRE3) to be quantified at thousands of individual metaphase kinetochores as they undergo poleward and anti-poleward motion. Surprisingly, we found that K-fibres are incoherent, containing both polymerising and depolymerising microtubules – with a small polymerisation bias for anti-poleward-moving kinetochores. K-fibres also display bursts of EB3 intensity, predominantly on anti-poleward-moving kinetochores, equivalent to more coherent polymerisation, and this was associated with more regular oscillations. The frequency of bursts and the polymerisation bias decreased upon loss of kinesin-13, whereas loss of kinesin-8 elevated polymerisation bias. Thus, kinetochores actively set the balance of microtubule polymerisation dynamics in the K-fibre while remaining largely robust to fluctuations in microtubule polymerisation.

## INTRODUCTION

Chromosome segregation requires that kinetochores form attachments to the plus-ends of parallel microtubule (MT) bundles called kinetochore-fibres (K-fibres) (McIntosh et al., 2012). In human cells, each K-fibre is ∼6 µm long and consists of 12–24 kinetochore MTs (K-MTs) (Compton, 2000; Rieder, 2005; Wendell et al., 1993); the K-fibre is dynamic, with plus-ends of K-MTs switching between polymerisation and depolymerisation. The half-life of a K-fibre is ∼200 s, an order of magnitude higher than free MTs within the spindle (Amaro et al., 2010; Zhai et al., 1995), indicating that K-MTs are under strong regulation, presumably by factors recruited to the kinetochore. Bi-orientated sister kinetochores (each sister is attached to a K-fibre emanating from opposite poles) can be in either a poleward-moving or away-from-the-pole (or anti-poleward)-moving state that is thought to be associated with the K-fibre being in a depolymerising or polymerising state, respectively. Sisters switch between these two states giving rise to the semi-periodic oscillations observed in prometaphase and metaphase, but are not perfectly coupled (Dumont et al., 2012; Jaqaman et al., 2010; Kitajima et al., 2011; Magidson et al., 2011; Skibbens et al., 1993; Stumpff et al., 2011; Wan et al., 2012). Although automated kinetochore tracking provides a quantitative description of kinetochore dynamics in human cells (Jaqaman et al., 2010; Vladimirou et al., 2013), we do not have a clear view of how these movements relate to the dynamics of individual K-MTs within the K-fibre.

The rapidity of kinetochore directional switching would seem to imply that the growth states of K-MTs within a given fibre are highly coordinated. In support of this idea are time-lapse microscopy experiments in PtK1 cells that report EB1 (also known as MAPRE1) predominantly associating with the anti-poleward-moving kinetochore (Tirnauer et al., 2002). Given that EB proteins track polymerising MT ends (Bieling et al., 2007; Stepanova et al., 2003), these data suggest that anti-poleward-moving kinetochores are bound to a polymerising K-fibre, whereas the poleward-moving kinetochores track a depolymerising fibre. By contrast, investigations using electron microscopy (EM) suggest that the dynamics of individual MTs within a K-fibre are not coordinated (VandenBeldt et al., 2006) thus questioning K-fibre coherence. However, because it is impossible to know whether the K-fibre and kinetochore being examined is moving anti-poleward or poleward, such EM studies only provide a snapshot of a K-fibre at a given time without the ability to correlate this information with kinetochore dynamics. Resolving these issues and determining how MT dynamics relate to kinetochore dynamics requires a live-cell microscopy approach. Here, we report an automated quantitative analysis which tracks kinetochore positions with high temporal resolution and then extracts the kinetochore-proximal EB3 (also known as MAPRE3) signal intensity. By analysing over 500 cells and 2500 kinetochores (see supplementary material Table S1) we provide the first comprehensive view of EB and kinetochore dynamics during directed motion in living human cells.

## RESULTS

**Fig. 1.**
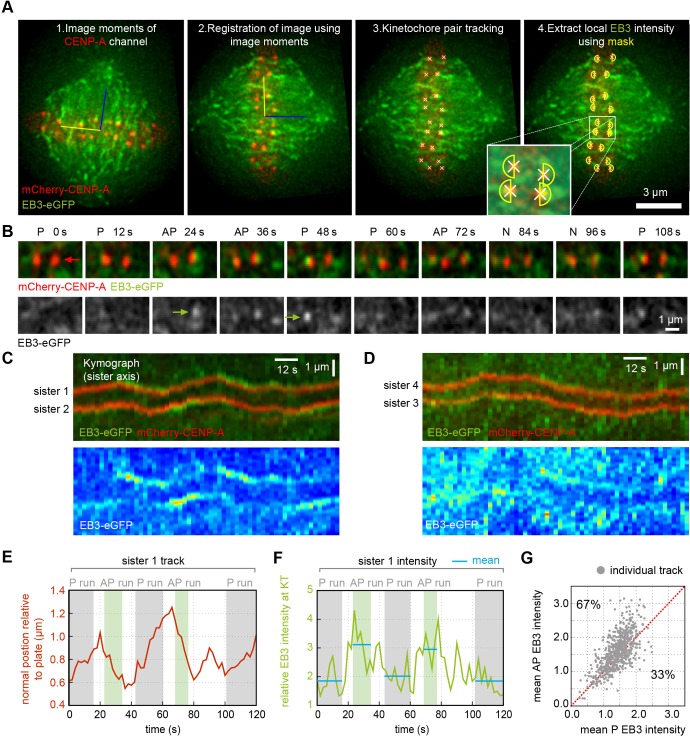
**EB3 preference for anti-poleward-moving kinetochores.** (A) 2D tracking of kinetochore positions (labelled with mCherry–CENP-A; red). 1: Image moments from the mCherry channel and centre of mass given by both channels define an (*x*,*y*) reference frame (blue and yellow lines). 2: Frames were registered using this coordinate system. The blue line is the spindle axis, yellow line is in the metaphase plate. 3: Kinetochore spots were located and tracked as described previously (Jaqaman et al., 2010). 4: Semi-circular masks (yellow) were centred on kinetochores orientated along the spindle axis allowing measurement of kinetochore-proximal EB3–eGFP intensity (green). (B) Individual frames from a typical mCherry–CENP-A EB3–eGFP movie in merged (top) and greyscale EB3–eGFP (bottom). The movement state of one kinetochore (red arrows) as poleward (P) or anti-poleward (AP) is given on top of each frame. N indicates directionless. EB3 labelling of the anti-poleward kinetochore is indicated by green arrows on greyscale image. (C,D) Kymographs of sister kinetochore pairs showing merged mCherry–CENP-A and EB3–eGFP (top) and false-colour EB3–eGFP (bottom). The kymograph profile is aligned along the sister–sister axis and is maintained at a fixed distance to the metaphase plate. This allows the visualisation of oscillations (see Materials and Methods), as opposed to kymographs that centre on the pair of kinetochores. (E) Kinetochore position relative to the metaphase plate versus time plot of sister 1 highlighted in C with anti-poleward (AP) runs shaded light green and poleward (P) runs shaded grey. Note that algorithm is conservative to avoid including directional switches where direction is uncertain. (F) Maximum EB3–eGFP intensity measured within the kinetochore mask (yellow semicircle in A) for sister 1 shown in C. Blue horizontal lines indicate means of each run. (G) Mean EB3–eGFP intensity of anti-poleward (AP) runs and poleward (P) runs within each track (*n*=850). Grey dots represent individual tracks. The red line indicates the mean EB3–eGFP intensity of anti-poleward runs being equal to mean EB3–eGFP intensity of poleward runs; the percentage of tracks above and below this line are indicated.

To examine EB and kinetochore dynamics, HeLa-Kyoto (HeLa-K) cells stably expressing mCherry–CENP-A (a marker for kinetochore position) and EB3–eGFP were filmed during metaphase, and single *z*-sections were captured every 2 s for 120 s in both the mCherry and eGFP channels (Fig. 1A; supplementary material Movie 1). As previously described, EB3 was enriched on the tips of polymerising astral and spindle MTs (presumably including kinetochore and non-kinetochore populations), as well as producing a weaker variable signal on the MT lattice (Komarova et al., 2009). To confirm EB3 was indeed labelling polymerising MTs in the spindle, we stabilised MTs with Taxol and found that EB3 delocalised from kinetochores and spindle MTs, becoming distributed throughout the cell (supplementary material Fig. S1A–C). Image sequences of single kinetochores showed occasional anti-poleward enrichment on the spindle pole side of the kinetochore (Fig. 1B; see also supplementary material Movie 2). By using a kymograph along the sister–sister axis, enrichment of EB3 during some anti-poleward runs was clearly observed (Fig. 1C). However, such events were variable as shown by the kymograph in Fig. 1D, taken from a cell of comparable overall brightness. Kymographs are useful for visualisation but they are inadequate for large-scale quantification because off-axis and dispersed signals are missed. We therefore measured the intensity of the EB3–eGFP signal in a mask centred on congressed single kinetochores (labelled with mCherry–CENP-A) over time (Fig. 1A; EB3 signals were normalised to allow for cell-to-cell comparison, supplementary material Fig. S1D; refer to Materials and Methods for details). Surprisingly, comparison of the kinetochore mask with equivalent masks in the spindle revealed that kinetochores were on average darker (supplementary material Fig. S1E). To relate the EB3 intensity to the direction of travel of the kinetochore, we classified each time step of each kinetochore track by direction (see Materials and Methods) – poleward (P), anti-poleward (AP) or where direction was uncertain, directionless (N) – thus automatically assigning sequences of poleward or anti-poleward runs within individual kinetochore tracks independently of its sister (Fig. 1E,F). Using the mean EB3–eGFP signal for each run we found the EB3–eGFP intensity to be greater on the anti-poleward-moving kinetochore in 67% of tracks (Fig. 1G, *P*<10^−24^, test details given in supplementary material Table S2 on row 1; we used α=0.01 as an indicator of strong statistical significance), where 50% would represent an absence of bias. Thus, EB3 is enriched on anti-poleward- compared to poleward-moving kinetochores more often than not, but this bias is far from consistent (33% had the reverse bias, see example in supplementary material Fig. S2A). This was not due to out of focus EB3 signal in our 2D imaging because the focal depth of the image was significantly larger than the size of the kinetochore (see Materials and Methods) and any movement of kinetochores in and out of focus would generate unbiased intensity variations (i.e. equally on anti-poleward- or poleward-moving kinetochores). We also calculated the average magnitude of the bias by taking the mean EB3 signal of all frames (from all tracks) that are in a poleward state and comparing them with all frames that are anti-poleward. This showed a limited but significant bias with the EB3–eGFP intensity being 12% higher in anti-poleward frames than in poleward frames (*P*<10^−67^, supplementary material Table S2, row 2). The anti-poleward bias was not dependent on the overall cell mean EB3 intensity (*P*=0.53, supplementary material Table S2, row 3). Our results thus contradict a simple model in which all MTs within a K-fibre are in either a coherent depolymerising or polymerising state. Instead, our live-cell data show that human K-fibres almost always consist of a mixture of MTs in both states.

**Table 1.**
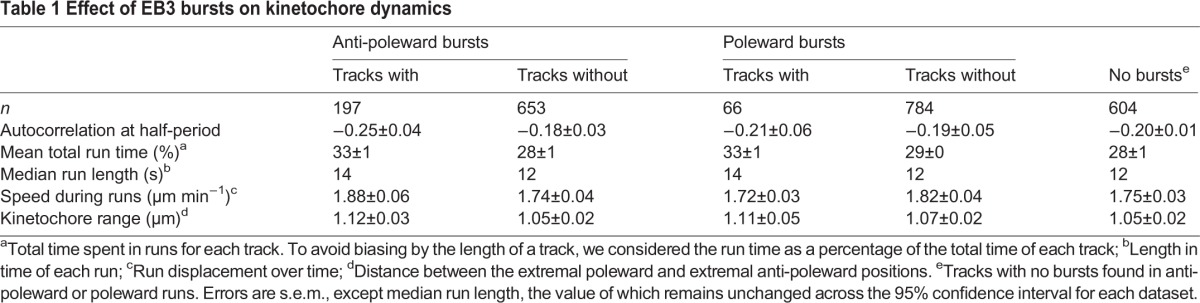
**Effect of EB3 bursts on kinetochore dynamics**

**Fig. 2.**
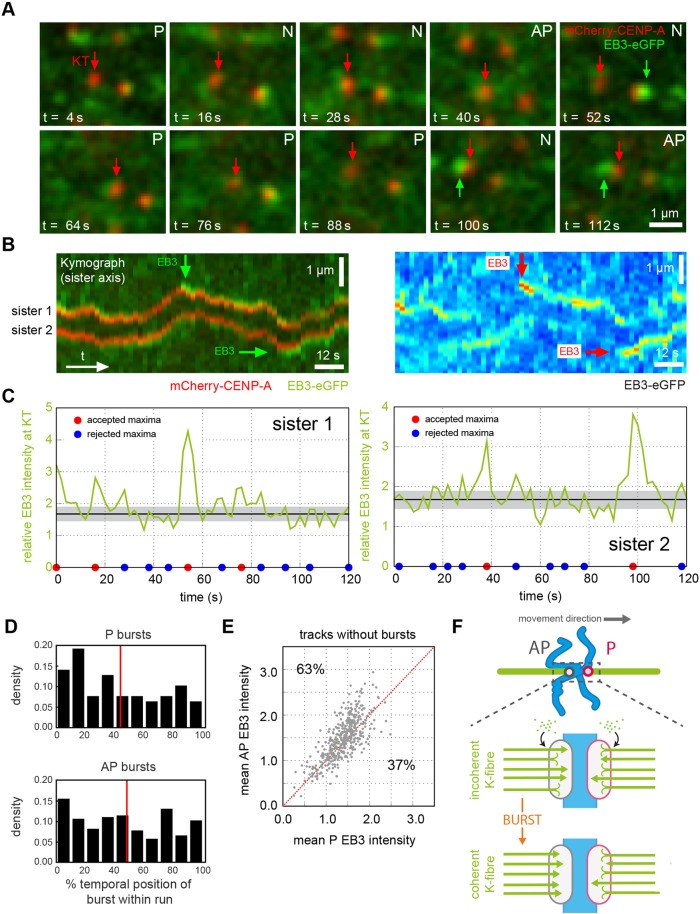
**Identifying kinetochores with EB3 bursts.** (A) Typical movie of a kinetochore with an EB3 burst. The red arrow indicates location of sister kinetochore 2 shown in B. The green arrow indicates an EB3 burst. P, poleward-moving kinetochore; AP, anti-poleward-moving kinetochore; N, directionless kinetochore. (B) Kymograph along the sister–sister axis of the kinetochore pair in A, showing EB3 bursts during anti-poleward runs (left). Same kymograph showing the EB3–eGFP channel in false colours (right). Red and blue indicate high and low intensity, respectively. (C) Maximum EB3–eGFP intensity signal within the mask of the kinetochore pair shown in B. Left panel, sister 1; right panel, sister 2. Large EB3 bursts occur at 54 s on sister 1 and at 38 s and 98 s on sister 2. Black line indicates mean EB3–eGFP signal excluding largest and smallest 10% of values, and shaded region indicates ±s.d. Local maxima in fluorescence were assessed for significance and the outcome of the test is shown at the base of each panel as accepted local maxima (red dot) and rejected maxima (blue dot). (D) Histogram of temporal position of poleward bursts (top; *n*=71) and anti-poleward bursts (bottom; *n*=248) within runs expressed as a percentage of run time to account for differing run lengths. The red line indicates mean burst time – 44% for poleward bursts (top) and 47% for anti-poleward bursts (bottom). (E) Mean EB3–eGFP intensity of anti-poleward runs and poleward runs within each track not containing an EB3 burst during any identified run within the track (*n*=604); grey dots represent individual tracks. The red line indicates the mean EB3–eGFP intensity of anti-poleward runs being equal to mean EB3–eGFP intensity of poleward runs; the percentage of tracks above and below this line are indicated. (F) Model of K-fibre during bursts. In normal circumstances (top) K-fibres from both sisters are in an incoherent polymerisation state. During a burst on the trailing (anti-poleward, AP) sister the majority of the MTs in this K-fibre become polymerising (bottom).

By inspecting the profiles of EB3 fluorescence at the kinetochore, we observed sudden and transient increases of 2–6 s duration in the EB3 signal (Fig. 2A, green arrows). We named these events bursts. A typical kinetochore with a burst during an anti-poleward run is shown as a kymograph in Fig. 2B with the EB intensity in Fig. 2C for the two sister kinetochores. We refer to a burst during an anti-poleward run as an anti-poleward burst; similarly for poleward bursts. The number of bursts found in kinetochore tracks was invariant with respect to the cell-to-cell variation in EB3–eGFP expression level, ruling out effects of protein overexpression or underexpression (supplementary material Fig. S2B; *P*=0.96, supplementary material Table S2, row 4). We further confirmed the EB3 burst phenotype in kymographs of a second cell line that expresses eGFP–CENP-A and EB3–tdTomato (supplementary material Fig. S2C).

Burst events occurred at higher frequency on anti-poleward kinetochores, with 23% of tracks having one or more anti-poleward bursts (*n*=197), whereas only 8% of tracks had one or more poleward bursts (*n*=66; *P*<10^−18^, supplementary material Table S2, row 5). There was no correlation between poleward and anti-poleward bursts occurring within the same track, indicating that we were not analysing a subset of kinetochores that are prone to bursts. The detection of poleward bursts was unexpected because the kinetochore-attached MTs are generally believed to be in a depolymerising state. One possible explanation is that poleward bursts reflect events immediately after a directional switch of the kinetochore: Salmon and colleagues have shown that at this moment the rate of poleward MT flux might maintain poleward movement of the kinetochore even though the MTs have switched to polymerisation (Wan et al., 2012). Our data provides weak support for this; we found a small enrichment of poleward bursts at the beginning of runs (Fig. 2D; partition 0–20% enriched versus 20–100%, *P*=0.009, supplementary material Table S2, row 6; anti-poleward bursts were more uniformly distributed). However, this hypothesis can only explain a small fraction of the poleward bursts because we observed poleward bursts throughout a run (Fig. 2D). Thus, we currently favour the idea that poleward-moving kinetochores can also switch their associated K-fibre into a transient near-coherent state, further supporting the idea that MT polymerisation is not limited to the anti-poleward-moving kinetochore. Furthermore, the distribution of burst intensity was very similar between poleward and anti-poleward bursts (supplementary material Fig. S2D). These data raise the possibility that there is no bias between poleward- and anti-poleward-moving kinetochores except when one sister undergoes a burst, that is, the 12% average increase in intensity of EB3 reported earlier might be due solely to the higher frequency of bursts on anti-poleward-moving kinetochores. To test this, we analysed tracks with no bursts and found that the anti-poleward-moving kinetochore was still favoured by EB3, although the preference was weaker (Fig. 2E; 63% compared to 67% when bursts are present as in Fig. 1G; *P*<10^−9^, supplementary material Table S2, row 7). The average magnitude of the anti-poleward bias in tracks without bursts was also reduced to 5% but was still significant (*P*<10^−11^, supplementary material Table S2, row 8). EB3 accumulation is thus heterogeneous with an intrinsic anti-poleward bias – presumably a constant labelling of anti-poleward-moving kinetochores – and transient bursts of EB3 of 2–6 s duration that are more frequent on anti-poleward- than poleward-moving kinetochores (schematic in Fig. 2F).

**Fig. 3.**
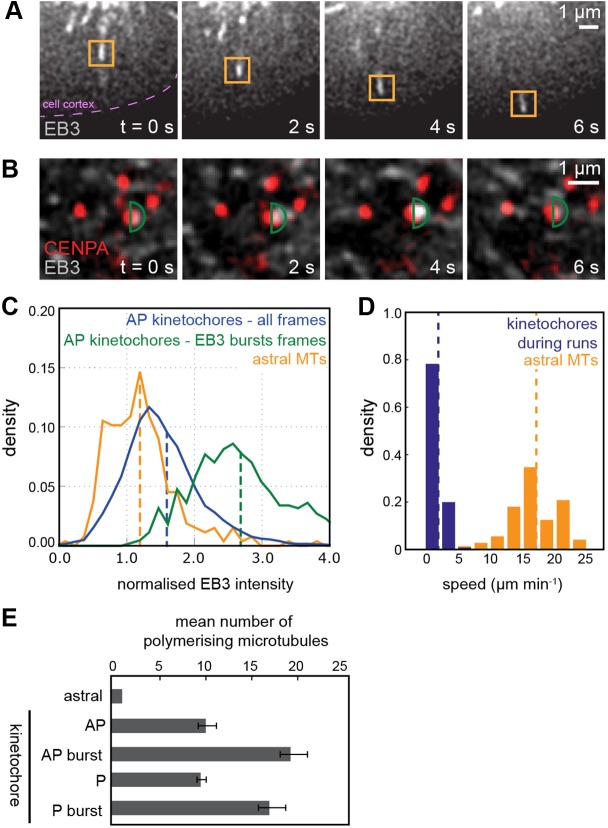
**Assessing degree of polymerisation in K-fibre by comparison to astral MTs.** (A) Growth of astral MT labelled with EB3–eGFP at the tip. The orange box indicates the mask used to measure maximum intensity of astral MT. Note that given that the MT is locally the brightest feature the size and shape of the mask is not important provided it encloses only the MT tip of interest. (B) Oscillating kinetochore pair labelled with mCherry–CENP-A (red) shown in overlay with EB3–eGFP (greyscale). The anti-poleward moving kinetochore (right sister of pair) experiences a burst in EB3–eGFP localisation at 4 s. Green semi-circle indicates fluorescent intensity measurement mask (radius 0.3 µm). The maximum pixel intensity within this mask is recorded. (C) Histogram showing intensity distribution of EB3–eGFP within the kinetochore mask (maximum pixel intensity within the mask is used), for all frames of anti-poleward kinetochores (blue) and during anti-poleward bursts (green; see B). The maximum mask intensity distribution of astral MT EB3 comets is shown in orange (see A). The average normalised EB3 intensities (indicated by dashed line) for astral MTs, anti-poleward kinetochores (all frames) and EB3 burst frames are 1.2, 1.6 and 2.7, respectively. (D) Kinetochore speeds (blue) during runs and astral MT speeds (orange) for growing astral MTs. The average speeds (indicated by dashed line) are 1.8 µm min^−1^ and 17 µm min^−1^ respectively (*n*=850 and 73, respectively). (E) Estimated mean number of polymerising MTs in anti-poleward (AP) and poleward (P) KTs and during anti-poleward (AP burst) and poleward bursts (P burst), compared to a single astral MT (see supplementary material Table S3 for details). Results represent mean±s.d. anti-poleward (AP, *n*=7134), anti-poleward with burst (AP burst, *n*=456), poleward (P, *n*=7058), poleward with burst (P burst, *n*=160).

In principle, we can estimate the number of polymerising MTs by comparing EB3 intensity to that of single astral MTs and scaling for the difference in speed. This is possible because as MT growth speed increases there is a proportional increase in EB3 associated with the tip (Bieling et al., 2007; Straube, 2011; Anne Straube, personal communication). We therefore measured the intensity and speeds of astral MTs (Fig. 3A) and compared the burst intensity measured from kinetochore tracks (Fig. 3B; see supplementary material Fig. S2D for distribution) to the intensity of single astral-MTs (Fig. 3C; see supplementary material Table S3 for intensities). To compensate for spindle fluorescence, we subtracted the mean kinetochore mask signal rather than the spindle signal, which is a poor estimate of background given that it is on average brighter than the kinetochore (supplementary material Fig. S1E; mean=0.36 from 50 cells). Bursts on anti-poleward runs have an average (mask maximum) intensity that is 2.2 times that of astral MTs (see Fig. 3C). We found the speed of kinetochores to be, on average, approximately ten times slower than astral MTs (Fig. 3D). The number of polymerising MTs was calculated as the ratio of kinetochore intensity to astral intensity, normalised for difference in speed: [(astral speed/kinetochore speed)×(mean of maximum kinetochore mask intensity−mean kinetochore mask intensity)]/(mean of maximum astral intensity). As a first approximation, we estimate that the average number of polymerising MTs at an anti-poleward-moving kinetochore is 10±2 and a burst increases the average to 19±3 (mean±s.d.; Fig. 3E; see supplementary material Table S3 for details). Given that K-fibres in metaphase from HeLa cells contain 12–24 (mean 17.1±0.6) MTs (Wendell et al., 1993), our data suggest that the burst reflects a high level of transient coherent polymerisation within the K-fibre.

**Fig. 4.**
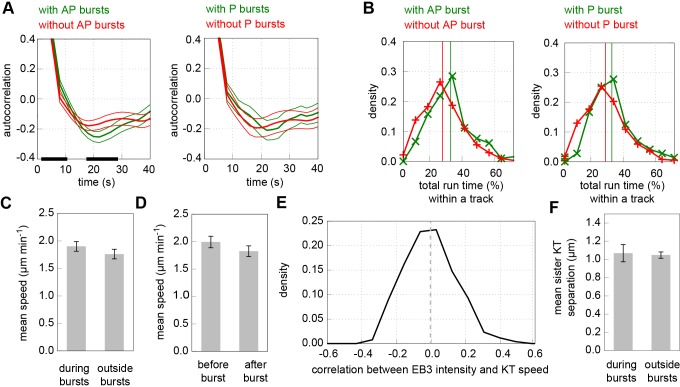
**Effects of bursts on kinetochore dynamics.** (A) Autocorrelation of kinetochore centre position displacement in direction normal to metaphase plate in tracks with (green, *n*=197) and without (red, *n*=653) anti-poleward (AP) bursts (left panel) and similarly for poleward (P) bursts (right panel; green, *n*=66; red, *n*=784). Shown are mean±s.e.m. autocorrelations. The black lines along the horizontal axis represent significant difference at the 1% level. (B) Total length of time spent in directed runs for each track as a percentage of track length, in tracks with (green, *n*=197) and without (red, *n*=653) an EB3 burst, in anti-poleward (left) and poleward bursting tracks (right; green, *n*=66; red, *n*=784). Vertical lines indicate means. (C) Mean kinetochore speed in a window of five frames around a burst (left; *n*=279) and mean from each run excluding the burst window (right; *n*=279). Error bars indicate s.e.m. (D) Mean kinetochore speed in 3 frames before the burst (left) and 3 frames after burst (right), not including the burst frame itself (*n*=319). Error bars indicate s.e.m. (E) Distribution of correlation between EB3–eGFP intensity and kinetochore speed (*n*=850). Dashed line indicates mean. (F) Mean separation between sister kinetochores in a window of five frames around a burst (left; *n*=112) and mean from each run excluding the burst window (right; *n*=112). Error bars indicate s.e.m.

To determine the function of EB3 bursts, we analysed the dynamics of kinetochores with and without bursts while they were undergoing poleward or anti-poleward runs. This analysis demonstrated that in the presence of anti-poleward bursts, oscillations were more regular (Fig. 4A; *P*<0.001, supplementary material Table S2, row 9), and there was a small difference in the total time spent in runs with either poleward or anti-poleward bursts (Fig. 4B; *P*<10^−5^ and *P*<0.001, respectively; supplementary material Table S2, rows 10, 11). The median individual run length was increased by one frame when containing a burst (*P*<10^−4^ and *P*=0.003 for anti-poleward and poleward bursts respectively; supplementary material Table S2, rows 12, 13); nevertheless, the change was so small we consider this inconclusive. However, the mean run speed (run displacement over time; *P*=0.06 and *P*=0.88 for anti-poleward and poleward bursts, respectively; supplementary material Table S2, rows 14, 15) and the range of each track (distance between the extremal poleward and anti-poleward positions, respectively; *P*=0.05 and *P*=0.26 for anti-poleward and poleward bursts, respectively; supplementary material Table S2, rows 16, 17) were unaffected (see Table 1 for averages). As the insensitivity of kinetochore speed to bursts was somewhat unexpected, we investigated this result further by looking for any changes in kinetochore speed during, before and after a burst (Fig. 4C,D; *P*=0.011 and *P*=0.19, respectively, supplementary material Table S2, rows 18, 19), and by correlating EB3 intensity with kinetochores speed (Fig. 4E; *P*=0.53, supplementary material Table S2, row 20). Each measurement showed no significant dependence on EB3. Despite anti-poleward bursts not causing a detectable speed change, they might still generate a pushing force; thus we might expect the anti-poleward-moving kinetochore to be pushed closer to the poleward kinetochore. However we did not observe any decrease in the sister–sister distance (Fig. 4F; *P*=0.87, supplementary material Table S2, row 21).

**Fig. 5.**
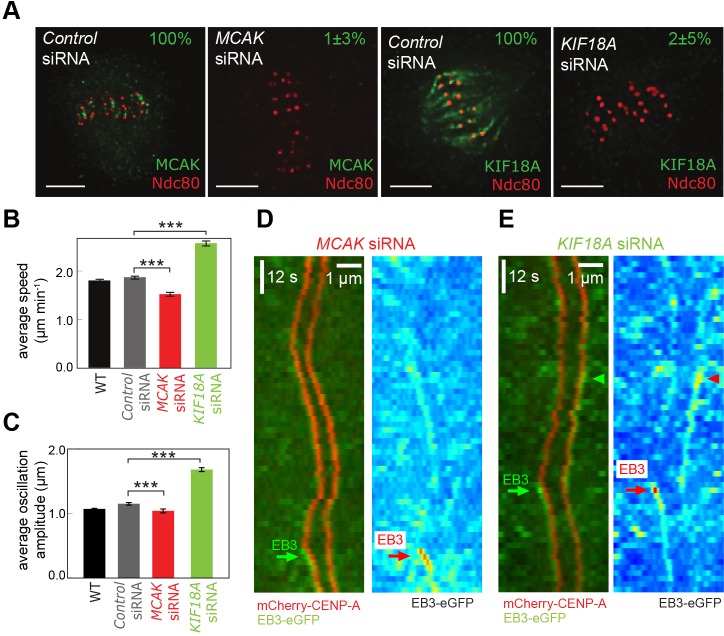
**The MT regulators MCAK and KIF18A influence EB3 localisation.** (A) Immunofluorescence images demonstrating siRNA depletion of MCAK and KIF18A. Percentage values quantify levels of depletion (mean±s.e.m., *n*=50). Scale bars: 5 µm. (B) Mean±s.e.m. (*n*=850, 850, 350, 469 respectively) kinetochore speed during runs for wild-type (WT), and control, *MCAK* and *KIF18A* siRNA-treated cells. (C) Mean±s.e.m. (*n*=850, 850, 350, 469 respectively) kinetochore oscillation amplitude for WT, and control *MCAK* and *KIF18A* siRNA-treated cells. ***P<0.001. (D) Kymograph of sister kinetochore pair in cells with MCAK depleted by siRNA, showing mCherry–CENP-A (red) and EB3–eGFP (green). Green arrows indicate strong EB3 localisation. The EB3–eGFP signal also shown in false colour. (E) As D, for cells with KIF18A depleted by siRNA.

**Fig. 6.**
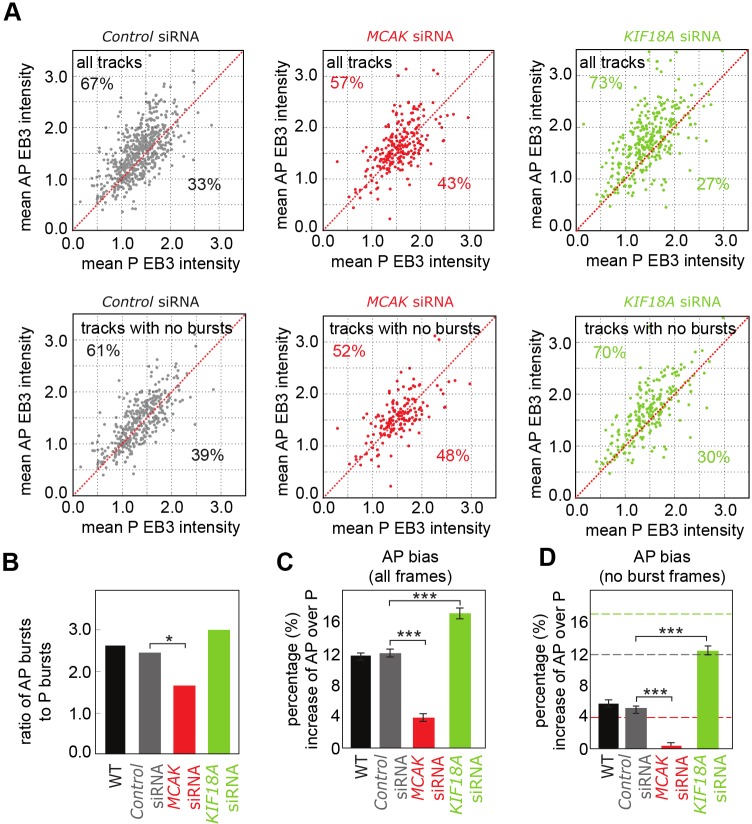
**Depletion of MCAK and KIF18A inhibits and enhances EB3 anti-poleward bias, respectively.** (A) Top, mean EB3–eGFP intensity of anti-poleward (AP) runs and poleward (P) runs within each track in control (left), *MCAK* (middle) and *KIF18A* (right) siRNA-treated cells (*n*=850, 350, and 469, respectively). Bottom, mean EB3–eGFP intensity of anti-poleward runs and poleward runs within tracks with no bursts in control (left), *MCAK* (middle) and *KIF18A* (right) siRNA cells (*n*=583, 262, and 352, respectively). The red line indicates the mean EB3–eGFP intensity of anti-poleward runs being equal to mean EB3–eGFP intensity of poleward runs; the percentage of tracks above and below this line are indicated. (B) Ratio of anti-poleward burst count to poleward burst count. WT, wild-type. (C) Bias of EB3 for anti-poleward kinetochore as a percentage increase over poleward kinetochore intensity (average across all frames). Results are the mean±s.e.m., see supplementary Table S2 rows 2, 29 and 36 for *n*. (D) Bias of EB3 for anti-poleward kinetochore as a percentage increase over poleward kinetochore intensity in tracks with bursts frames excluded. Results are the mean±s.e.m., see supplementary Table S2 rows 8, 30 and 37 for *n*. Dashed lines indicate the bias of frames in all tracks for comparison for control (grey), *MCAK* siRNA (red), *KIF18A* siRNA (green) from (C). *P<0.05; ***P<0.001.

So far, these data suggest that kinetochore dynamics are largely robust to transient fluctuations in MT polymerisation while at the same time the number of polymerising MTs appears to be kept low with a small intrinsic anti-poleward bias. This suggests that the kinetochore must have mechanisms to regulate and coordinate the number of polymerising MTs in a K-fibre. Multiple proteins influence K-MT dynamics (Cheeseman and Desai, 2008) and are therefore candidates for setting up the anti-poleward bias of EBs and/or inducing coherence (bursts). We investigated MCAK (also known as KIF2C) and KIF18A (members of the kinesin-13 and kinesin-8 families, respectively), two kinesin motor proteins that localise to kinetochores and influence MT dynamics but do not abolish oscillations (Jaqaman et al., 2010; Stumpff et al., 2008), which are needed to ascertain directed motion towards and away from their attached pole. We first depleted MCAK, which functions to promote MT catastrophe (Desai et al., 1999; Hunter et al., 2003; Moore et al., 2005), by small interfering RNA (siRNA) treatment using two independent siRNA oligonucleotides (see supplementary material Table S4 for results with the second siRNA), and confirmed loss of the protein from kinetochores by immunofluorescence (Fig. 5A). The overall EB3 fluorescence was consistent with control cells and the observed drop in kinetochore speed and oscillation amplitude (Fig. 5B,C; *P*<10^−9^ and *P*<0.001, respectively supplementary material Table S2, rows 22, 23) was in agreement with previous work (Jaqaman et al., 2010). Kymographs from cells depleted of MCAK appeared to show less obvious bias of EB between anti-poleward- and poleward-moving kinetochores (Fig. 5D), although bursts could still be observed (Fig. 5D, green arrow). We confirmed that burst frequency in the control and *MCAK* siRNA cells was not sensitive to overall cell fluorescence (supplementary material Fig. S3; *P*=0.32 and *P*=0.37, respectively; supplementary material Table S2, rows 24, 25). Depletion of MCAK resulted in a reduction of the fraction of tracks with EB3 bias to anti-poleward-moving kinetochores (57%) compared to control (67%; Fig. 6A, upper panel; *P*=0.008; supplementary material Table S2, row 26). The depletion also directly affected burst frequency, reducing the excess of anti-poleward to poleward bursts, the ratio having a weakly significant fall from 2.5 to 1.6 with *MCAK* siRNA (Fig. 6B; *P*<0.04, supplementary material Table S2, row 27). One possibility is that this reduction in burst frequency explains why the anti-poleward bias is reduced. To test this, we repeated the analysis but excluded any tracks where there was a burst. Strikingly, the number of tracks with higher EB3 on anti-poleward kinetochores was reduced to insignificant levels, 52%, compared to 61% in control cells, when restricted to tracks without bursts (Fig. 6A, lower panel; *P*=0.41, supplementary material Table S2, row 28). This indicates that loss of MCAK activity not only reduces bursts but also eliminates the intrinsic bias between poleward- and anti-poleward-moving kinetochores. To confirm this, we compared the mean intensity of EB3–eGFP in all anti-poleward and poleward frames. This analysis showed that the bias to the anti-poleward kinetochore was 12% and was reduced to 4% when MCAK was depleted (Fig. 6C; *P*<10^−7^, supplementary material Table S2, row 29). Furthermore, the intrinsic bias (observed by excluding frames with a burst) was eliminated (Fig. 6D; *P*=0.39; supplementary material Table S2, row 30). We therefore conclude that MCAK is essential for maintaining the normal level of intrinsic bias of EB proteins for the anti-poleward-moving kinetochore, while also increasing the frequency with which the K-fibre attains coherence. It is important to note that the loss of bias does not mean that there are no polymerising MTs on the anti-poleward-moving kinetochore, hence oscillations are still possible.

KIF18A was originally reported to be a MT depolymerase (Mayr et al., 2007), although recent work indicates that it has a more general effect in suppressing MT dynamics (Du et al., 2010; Stumpff et al., 2008). Depletion of KIF18A was also efficient (Fig. 5A), and reproduced the previously reported effect on kinetochore speed and oscillation amplitude (Fig. 5B,C; *P*<10^−36^ and *P*<10^−60^; supplementary material Table S2, rows 31, 32; see supplementary material Table S4 for results with the second siRNA) (Jaqaman et al., 2010; Stumpff et al., 2008; Wordeman et al., 2007). Again, we confirmed that burst frequency was not sensitive to overall cell fluorescence (supplementary material Fig. S3; *P*=0.26, supplementary material Table S2, row 33). KIF18A depletion had a distinct effect from MCAK on polymerisation dynamics: the ratio of anti-poleward to poleward bursts appeared to increase to 2.9, but this was not statistically significant (Fig. 6B; *P*=0.11; supplementary material Table S2, row 34). However, the number of tracks with higher EB3 on the anti-poleward-moving kinetochore was also increased to 73% (Fig. 6A; *P*<0.001; supplementary material Table S2, row 35) – this was evident in kymographs of kinetochore pairs (Fig. 5E). Furthermore, an analysis across frames indicated that the bias to an anti-poleward-moving kinetochore was increased to 17% (Fig. 6C; *P*<10^−6^, supplementary material Table S2, row 36) and, by excluding burst frames, the intrinsic anti-poleward bias (with burst frames excluded) was still present at 13% – significantly higher than in control cells (Fig. 6D; *P*<10^−8^; supplementary material Table S2, row 37). Thus, the KIF18A motor is specifically required at kinetochores to reduce polymerisation coherence in the K-fibre and inhibit bursts.

## DISCUSSION

The small and infrequent EB3 bias to the anti-poleward-moving kinetochore observed here indicates that K-fibres are not in an highly coherent polymerisation state. Our findings in live-cells suggest that the typical human K-fibre is predominantly incoherent, containing polymerising MTs whether it is moving poleward or anti-poleward. This is consistent with experiments demonstrating that all metaphase kinetochores incorporate tubulin into their attached K-fibre (Mitchison et al., 1986) and with the end structure of the individual tips as revealed by EM tomography of kinetochores in Ptk1 cells, showing a mixture of polymerising and depolymerising K-MTs (VandenBeldt et al., 2006). The previously proposed ‘binary model’, in which MT polymerisation is restricted to the anti-poleward kinetochore (Tirnauer et al., 2002) is therefore incorrect for most kinetochores – although we cannot rule out differences between cell types. We suggest that the apparent inconsistency with these previous live-cell experiments reflects the limitations of qualitative analysis of a small population of kinetochores. Indeed, from the scatter plot in Fig. 1G it is evident that some kinetochore tracks do show a strong bias (see below) although the overall bias in the population is low. Our conclusions are compatible with mathematical models of chromosome oscillations in which the K-fibre comprises multiple highly dynamic K-MTs each of which can switch independently between growth and catastrophe (Civelekoglu-Scholey et al., 2006; Joglekar and Hunt, 2002). These models are capable of producing directed steady motion in certain conditions suggesting that a mechanism to enforce coherence in the K-fibre is not essential. However, the absence of a strong bias raises important mechanistic questions as to how a poleward-moving kinetochore is able to move when attached to polymerising MTs. One idea is that the kinetochore can operate as a clutch and disengage from individual (polymerising) K-MTs leaving them in a non-force generating state. This mechanism would also require that the kinetochore maintains a grip on depolymerising MTs within the K-fibre and this would generate driving force.

Our data also reveals that kinetochores can experience bursts of EB3 signal for short durations (2–6 s) that represent near full coherence of MT polymerisation. Surprisingly, bursts had rather small effects on kinetochore dynamics (although oscillations were more regular) suggesting that the kinetochore is relatively insensitive to large fluctuations in the numbers of polymerising MTs. Moreover, the short duration of these events imply that kinetochores actively inhibit the coherent state. This might be important to avoid the complication of having to coordinate all of the ∼12–24 K-MTs within a given K-fibre in order to generate chromosome motility.

Importantly, by perturbing the regulation of MT polymerisation dynamics using siRNA-mediated protein depletions, we confirm that EB3 signals truly reflect polymerisation dynamics *in vivo*. Surprisingly, the depletion of MCAK – a MT depolymerase from the kinesin-13 family (Wordeman et al., 2007) – eliminated the intrinsic EB bias between poleward- and anti-poleward-moving kinetochores and reduced the frequency of bursts. This implies that both sisters have similar numbers of polymerising MTs (in the absence of bursts, which have an anti-poleward bias). The fact that oscillations are intact under these conditions again demonstrates that kinetochores are very robust to changes in the number of polymerising MTs, and supports the idea that the depolymerising MTs attached to the poleward-moving kinetochore are sufficient for oscillations. We do not yet know how MCAK controls the EB intrinsic bias. However, we speculate that its loss from the poleward-moving kinetochore would cause MTs within this K-fibre to have a higher probability of being in a polymerising state. As well as eliminating the EB bias, this would also be expected to reduce driving forces (depolymerisation-coupled pulling), thus providing a potential explanation for the reported slowing of kinetochore movement in MCAK-depleted cells (Jaqaman et al., 2010; Wordeman et al., 2007).

KIF18A appears to have the opposite effect: depletion of the motor increases speeds and polymerisation bias. This would suggest that the KIF18A motor is functioning to reduce K-MT polymerisation. Consistent with this, biochemical experiments show that KIF18A is able to cause MT polymerisation to pause upon reaching the MT plus-end (Du et al., 2010; Stumpff et al., 2012). Because the motor is highly processive (Mayr et al., 2011; Stumpff et al., 2011), the current models propose that KIF18A accumulates at kinetochores in a MT-length-dependent manner and increases the probability of a directional switch, presumably by inhibiting polymerisation and thus increasing the probability that MTs attached to the anti-poleward-moving kinetochore undergo catastrophe (Stumpff et al., 2008; Stumpff et al., 2012). A direct prediction of this model is that depletion of KIF18A would increase the number of polymerising MTs and/or the speed of polymerisation: either event should increase the EB signal on anti-poleward-moving kinetochores. Our data provides a confirmation of this model through direct visualisation of the polymerisation dynamics at kinetochores. We suspect that the increased speeds are the result of there being fewer depolymerising MTs on the anti-poleward-moving kinetochore, which would otherwise slow the progress of the sister pair. We note that absence of a correlation between the natural variations in kinetochore speed in wild-type cells (Fig. 4C–E) and MT polymerisation (EB accumulation) simply reflects the greater magnitude of speed changes caused by depleting KIF18A or MCAK.

This study highlights the power of combining object tracking and intensity measurements to relate the dynamics of cellular structures to their biochemical states. We propose that the K-fibre is a dynamic system under the control of the kinetochore, with fewer or greater than equal numbers of polymerising and depolymerising MTs. Kinetochore-bound MCAK and KIF18A appear to operate antagonistically with respect to regulating the balance of these K-fibre MT dynamics. The largely incoherent nature of K-fibre polymerisation dynamics might provide a mechanism by which the kinetochore can regulate speed and allow for rapid switches of direction, while maintaining a robust attachment to the K-fibre.

## MATERIALS AND METHODS

### Cell culture, siRNA transfection and drug treatment

To establish the stable HeLa-K cell line expressing mCherry–CENP-A EB3–eGFP, an eGFP–CENP-A plasmid (pMC167) was first modified by exchanging the eGFP for mCherry using the *Nhe*I and *Eco*RI sites. This plasmid (pMC303) was transfected using Fugene (Promega, Southampton, UK) into Hela-K cells and single clones were selected with 0.3 µg ml^−1^ Puromycin (MC051). MC051 cells were then transfected with a mouse EB3–eGFP construct (kind gift from Anne Straube, Warwick Medical School, University of Warwick, UK) and single clones selected with 500 µg ml^−1^ G418 (MC068). We used our existing kinetochore tracking assay (Jaqaman et al., 2010) to confirm in 3D tracks that the half-period of sister kinetochore oscillations remained at ∼35 s as previously described. HeLa-K mCherry–CENPA EB3–eGFP (MC068) cells were cultured in Dulbecco's modified Eagle's medium (DMEM) containing 10% FBS, 1% penicillin-streptomycin, 0.3 µg ml^−1^ puromycin and 500 µg ml^−1^ G418, and HeLa-K mCherry–CENP-A (MC051) were cultured in DMEM containing 10% FBS, 1% penicillin-streptomycin and 0.3 µg ml^−1^ puromycin. eGFP–CENP-A EB3–tdTomato cells were handled as previously described (Samora et al., 2011). All cell lines were grown at 37°C with 5% CO_2_. siRNA-mediated interference oligonucleotides were: control siRNA (Samora et al., 2011), *MCAK* siRNA [oligonucleotide 1, GAUCCAACGCAGUAAUGGU (Cassimeris and Morabito, 2004), oligonucleotide 2, GGGCAGACA-UUUGCCAACUCCAAUU (Invitrogen, Paisley, UK)] and *KIF18A* siRNA [oligonucleotide 1, CCAACAACAGUGCCAUAAA and oligonucleotide 2:, ACAGAU-UCGUGAUCUCUUA) (Mayr et al., 2007)] were transfected into HeLa-K mCherry–CENP-A EB3–eGFP cells (MC068) using Oligofectamine (Invitrogen, Paisley, UK) as per the manufacturer's instructions and imaged 48 h after transfection. siRNA treatments were validated by quantitative immunofluorescence. For the MT stabilisation experiments, cells were treated with 10 µM Taxol (Tocris Bioscience) for 1 h prior to live-cell imaging.

### Live cell imaging and kinetochore tracking

Live-cell imaging experiments were performed at 37°C with 5% CO_2_ in FluoroDish tissue culture dishes with cover glass bottoms (WPI). Dual-colour imaging of HeLa-K mCherry–CENPA EB–eGFP cells for kinetochore or EB3 comet tracking was carried out on a Deltavision Elite microscope system equipped with a 100× oil NA 1.4 objective. A single plane was recorded every 2 s for 120 s using a 100 ms exposure, 50% neutral density in GFP (475/28 filter) and mCherry (575/25 filter), using high-speed emission filters for hsGFP (525/50 filter) and hsmCherry (632/60 filter) and the dichroic mirror Quad-mChe-Hs. Movies were deconvolved (2D) with a constrained iterative deconvolution algorithm with a medium noise filtering and eight iterations using SoftWorx (Applied Precision). There is a compromise between imaging at high speed in 2D, thereby potentially losing kinetochores from the focal plane, and imaging slowly in 3D but tracking more completely. Here, we chose high-speed 2D imaging to improve resolution of runs and, as a consequence, sometimes lose track information. However, due to the depth of the focal plane, we can measure the maximum intensity pixel of a tracked spot for a substantial portion of a movie. Moreover, we confirmed EB3–eGFP intensity was poorly correlated with mCherry–CENP-A intensity (correlation coefficient=0.14±0.01, ±s.e.m.) and analysis of the latter revealed no differences between poleward- and anti-poleward-moving kinetochores indicating that EB3–eGFP dynamics were not due to spot defocusing. Spot tracking of mCherry-labelled kinetochores was performed using the KiT (Kinetochore Tracking) software, evolved from the previously described MaKi software (Jaqaman et al., 2010) (KiT is available for download from http://www.mechanochemistry.org/mcainsh/software.php). The coordinate system used in the original version of the software, which relied on the 3D distribution of kinetochores, proved too noisy for our 2D data so we defined a coordinate system based on the image moments of the mCherry channel, filtered to accentuate the kinetochores. Image moments are weighted averages of the pixel intensity,

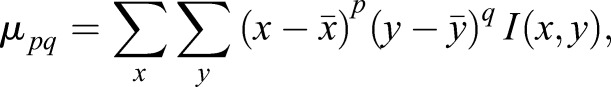


where *I*(*x*,*y*) is the pixel intensity at location (*x*,*y*). The image covariance matrix describes the orientation of the image,

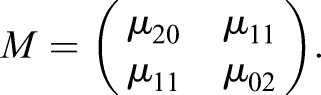


The eigenvector associated with the largest eigenvalue of *M* defines the direction with the largest variance. This eigenvector lies parallel to the metaphase plate; the other is used as the normal axis. The origin of the coordinate system was defined as the centre of mass of an equal weighted blend of both mCherry and eGFP channels.

### Intensity measurement

Fluorescence intensity values were extracted directly from the raw image data per kinetochore per frame. We masked out the whole frame except for a semi-circle centred at the position of the tracked kinetochore (mCherry–CENP-A). We used the maximum intensity within this mask within each frame to capture the peak of the diffraction-limited spot formed by EB3 at MT ends (K-fibres change length too slowly to form comets such as those seen on astral-MTs). These diffraction limited spots were only occasionally resolvable – because of the high levels of noise from the spindle and fluctuating EB3 signal at the KT spot, tracking techniques had poor efficacy, which limited quantification by this method. Quantification in a mask overcame this problem. The arc of the semi-circular mask is directed toward the pole to which the kinetochore is attached (see Fig. 1A). We also examined circular and ‘Pac-man’-shaped masks and various radii. These did not qualitatively affect the results. Based on electron (DeLuca et al., 2005) and fluorescence (Wan et al., 2009) microscopy, K-MTs terminate in the outer kinetochore plate, between 40 and 100 nm from CENP-A, so we used a radius of 300 nm to adequately cover the kinetochore, yet avoid inclusion of non-kinetochore MTs in an overly large mask. A range of descriptive statistics for the extracted pixels were recorded from each kinetochore mask per frame. Fluorescence intensity values were corrected for photobleaching before any further analysis. The mean frame intensity curve was well-fitted by a double exponential, and photobleaching correction was performed by rescaling the photobleaching profile such that the first frame has mean 1, and then dividing the intensities from each frame by the fitted double exponential curve. After photobleaching correction, intensities were normalised per cell, thus allowing comparison of signals from different cells using the cytoplasmic and spindle background separately (see supplementary material Fig. S1D); simple background subtraction would not account for fluorescence variation between cells and variable levels of transformation efficacy. We used *k*-means clustering to sort all pixels from the whole image into two groups – spindle and cytoplasmic background – taking the brightest to be the spindle. Denoting the centroids of the spindle and cytoplasm *I_s_* and *I_c_* (see supplementary material Fig. S1E), respectively, normalised intensities *I′* were calculated from raw intensities *I* by 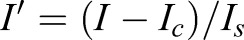, that is, the spindle has mean normalised intensity 1 and the cytoplasm has mean normalised intensity zero in each cell.

### Run direction assignment

Kinetochore displacements were computed and then standardised such that a positive displacement means a poleward movement and a negative displacement means an anti-poleward movement. Owing to the noisiness of kinetochore tracks, taking the sign of the displacement was not sufficient to determine consistent movement in one direction. We therefore devised an algorithm for identifying direction based on neighbouring displacements. Each kinetochore was treated independently and each frame was given a score based upon how many poleward or anti-poleward moving frames there were within a window of size *w* around that frame,

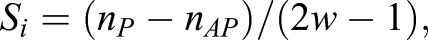


where *n_P_* and *n_AP_* are the number of poleward and anti-poleward moving frames in the window, respectively. Frames with S_i_≥−S* were assigned the label poleward, and those with S_i_≥S* were assigned the label anti-poleward. We found S*=0.25 and *w*=7 to be effective. Frames not meeting either criterion were considered directionless. The algorithm is conservative in the sense that frames close to a switch in direction are not included in a run (see excluded regions in Fig. 1E,F).

To identify segments of tracks with persistent movement in one direction – we called these runs – we located sets of *L* consecutive frames with the same label, either poleward or anti-poleward, where 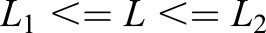 (see example in Fig. 1E). We found the criteria 
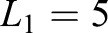
 and 
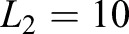
 to be reasonable, corresponding to runs of 10 to 20 s. To ensure that only kinetochores making directed movements, as opposed to random diffusion, contributed to statistics, we filtered out all tracks that did not have at least one run of 10–20 s. A maximum run length was imposed to exclude tracks that drifted in one direction throughout; typically these did not exhibit oscillations and their movement was likely driven by different processes.

### Burst identification

We identified EB3 bursts by searching for peaks in the fluorescence intensity by using the following algorithm: Denote the intensity signal during the run as *I(t)*, where *t* is the set of discrete time points at which the signal is measured. Let *I_D_(t)* be the result of a maximum filter of window size *w* applied to *I(t)*. The location of local maxima are then the points such that *I(t)−I_D_(t)*=0. Then estimate the background mean and variance from the remaining signal, that is the non-local maxima points. Estimate the signal mean and variance from a window of size *w* around each maximum. Test each maximum against the background using a *t*-test, assuming equal variance against a threshold α/*n*, where *n* is the number of maxima [division by *n* applies the Bonferroni correction for multiple testing to reduce false positive rate (Hochberg and Tamhane, 1987)], to accept maxima at the 1−α significance level. In this study, we have used *w*=5 and α=0.01. The assumption of equal variance for the *t*-test was justified by an F-test, which failed to reject the null hypothesis of equal variances.

### Hypothesis testing

All hypothesis tests were standard, with full details of each test given in supplementary material Table S2, except for testing autocorrelations which is described here. To test for a significant difference between samples of autocorrelations, we used a random permutation test. For the samples to be tested, we computed as test statistics the difference between the amplitude of the oscillation and the difference between the half-period. We also used the difference at each time lag as a statistic. We then permuted the labels of individual observations between the two samples and recomputed the test statistics. Repeating this procedure many times estimates the distribution of the test statistic; a *P*-value is then derived from the fraction of iterations where the test statistic exceeded that of the original samples.

### Metaphase plate referenced kymograph

Kymographs are a sequence of line intensity profiles, one for each frame of a movie. To generate kymographs from movies with non-stationary structures, it is necessary to obtain a reference point to fix the line profile for each frame. Without a reference point the structure of interest will often move out of the profile. Existing tools for generating kymographs of kinetochores use one of the sisters or their centre as a reference point (Pereira and Maiato, 2010). Consequently they are unable to observe overall motion of the pair – only relative motion between them. Thus, we developed a new scheme using the metaphase plate as a reference (supplementary material Fig. S4). For the first frame, a line of fixed length *l* was drawn passing through the two sister kinetochores *s*_1_ and *s*_2_, with midpoint equidistant from both and extending beyond the sisters in both directions. The end points of the line we denote *p*_1_ and *p*_2_. The distance *d* between the metaphase plate plane and the kymograph line was held constant from frame to frame according to the following scheme:

The metaphase plate defines a plane with equation

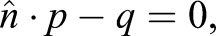


for points *p* in the plane. For the point *p*_1_, which is not in the plane in general, the distance to the plane *d* is given by

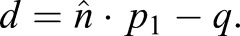


Let the normalised inter-sister vector be

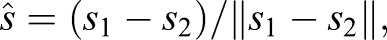


where 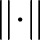 indicates the length of a vector, then

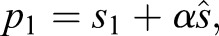


where α is a scalar, that is *p*_1_ is on the line passing through the sister kinetochores. To find α, and hence where to locate the kymograph line, substitute for *p*_1_ in the equation for *d* above. Solving, we find

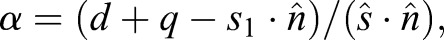


where the only free parameter is *d*. We chose to fix *d* in the first frame such that

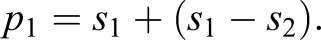


This results in a kymograph profile twice the length of the initial distance between the sisters but with a fixed distance from the metaphase plate. Therefore, the profile position is invariant to kinetochore oscillations, rendering them observable in the kymograph, and furthermore takes advantage of the kinetochore tracking to remain aligned over the pair.

## Supplementary Material

Supplementary Material
