# Supplementary material for: Probing microtubule polymerisation state at single kinetochores during metaphase chromosome motion
Source: J Cell Sci. 2015 May 15;128(10):1991–2001. doi: 10.1242/jcs.168682 (PMC4457160; doi:10.1242/jcs.168682)
Supplement: Supplementary Material [file supp_128_10_1991__index.html]

Probing microtubule polymerisation state at single kinetochores during metaphase chromosome motion — Supplementary Material 

# Probing microtubule polymerisation state at single kinetochores during metaphase chromosome motion

## JCS168682 Supplementary Material

**Files in this Data Supplement:**

- **Supplementary Material**
