# Supplementary material for: Probing microtubule polymerisation state at single kinetochores during metaphase chromosome motion
Source: J Cell Sci. 2015 May 15;128(10):1991–2001. doi: 10.1242/jcs.168682 (PMC4457160; doi:10.1242/jcs.168682)
Supplement: Supplementary Material [file supp_128.10.1991_JCS168682.pdf]

Supplementary Information for Probing microtubule polymerisation state at single kinetochores during metaphase chromosome motion

Jonathan W. Armond, Elina Vladimirou, Muriel Erent, Andrew D. McAinsh, Nigel J. Burroughs

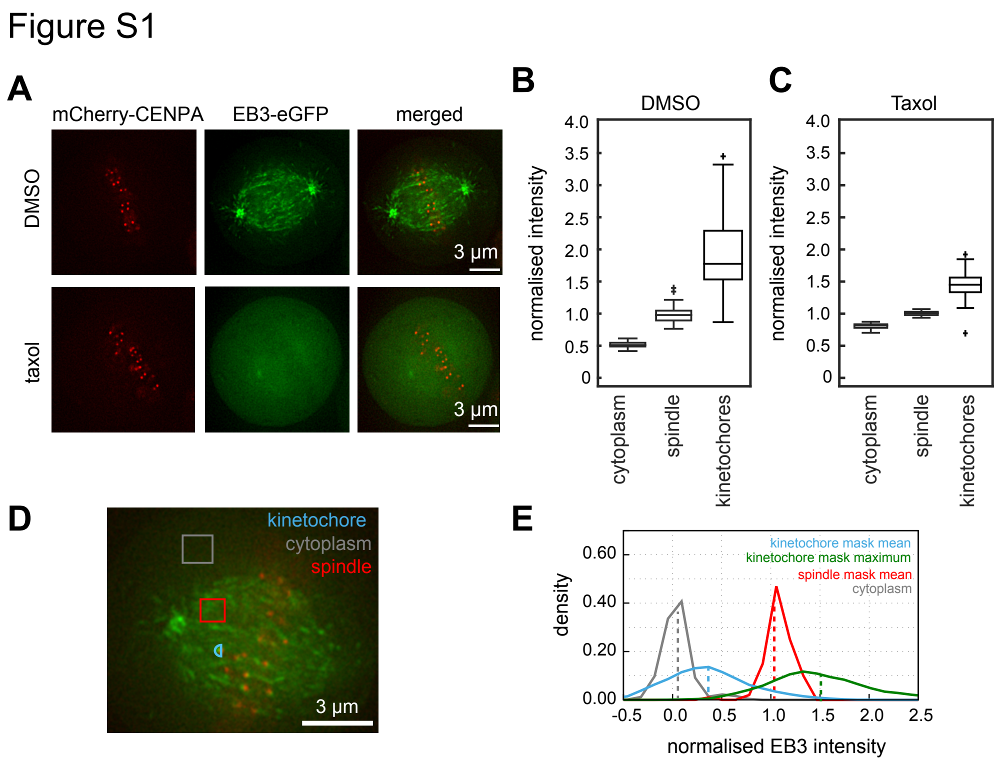

Figure S1. Stabilisation of microtubule dynamics and intensity normalisation.
(A) Taxol treatment redistributes EB3 to the cytosol. Left to right: mCherry-CENP-A red channel, EB3-eGFP green channel, and merged. Top: Control cell treated with DMSO. Bottom: Cell treated with taxol. The intensity scale is identical in top and bottom panels for each channel.
(B) Boxplot of maximum intensity (rescaled per cell such that spindle mean is 1) in semi-circular masks at several locations in the cytoplasm, spindle and at the kinetochore (KT) for DMSO treated cells ($n_{DMSO\ spindle}$=26, $n_{DMSO\ KT}$=805; 5 cells).
(C) As in (B) for Taxol treated cells ($n_{Taxol\ spindle}$=30, $n_{Taxol\ KT}$=519; 5 cells).
(D) Indicative locations of masks for measuring intensities within the cytoplasm (grey), spindle (red) and at the kinetochore (blue; semi-circular mask).
(E) Normalised histograms of mean (except for kinetochore where maximum is also shown in green) EB3-eGFP fluorescence intensity within the indicative masks drawn in (D) for 50 cells. Dotted lines indicate histogram means.

Figure S2

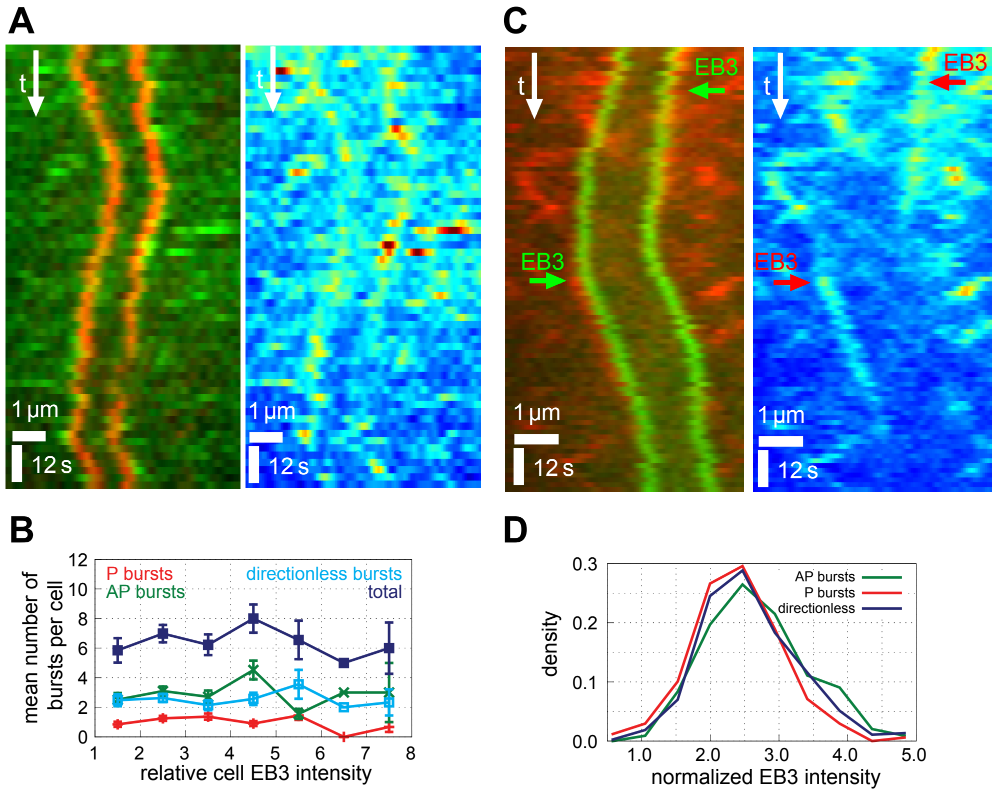

Figure S2. Confirmation of burst phenotype and distribution.

(A) Kymograph of an oscillating kinetochore pair with the left kinetochore having higher EB3-eGFP intensity during P runs than AP (left panel). Kymograph is reproduced in false colour for EB3-eGFP channel (right).

(B) Mean number of bursts per cell found in P runs (red), AP runs (green), not in runs (cyan) and the total number of bursts (blue) binned according to relative mean signal intensity of EB3-eGFP in mCherry-CENP-A/EB3-eGFP cells after background subtraction. The intensity scale is relative to the least bright cell in the dataset. We observed a five-fold range of relative fluorescence intensity, indicating the variability in EB3 overexpression. Error bars indicate s.e.m.

(C) Kymograph of an oscillating kinetochore pair with sporadic labelling of AP KT by EB3 in eGFP-CENP-A/EB3-tdTomato cell line (indicated by arrows). An EB3 burst is visible after approximately 60 s. Kymograph is reproduced in false colour for EB3-tdTomato channel (right; blue/red indicate low/high intensity).

(D) Distribution of burst peak fluorescent intensities, categorised into P, AP and directionless bursts.

Figure S3

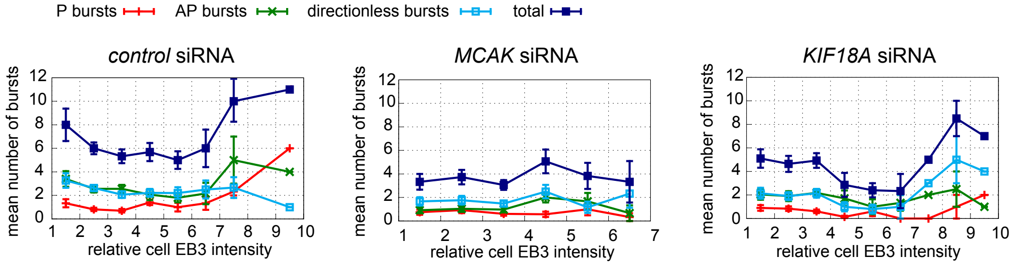

Figure S3. Verifying independence of burst frequency from cell brightness.
Mean number of bursts per cell for *control, MCAK* and *KIF18A* siRNAs (left, middle, right respectively) found in P runs (red), AP runs (green), not in runs (directionless; cyan) and the total number of bursts (blue) binned according to relative mean signal intensity of EB3-eGFP in mCherry-CENP-A/EB3-eGFP cells after background subtraction. The intensity scale is relative to the least bright cell in the dataset. We observed a similar range of fluorescence intensities for each dataset. Error bars indicate s.e.m..

Figure S4

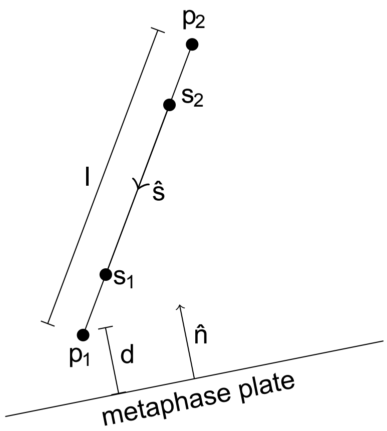

Figure S4. Kymograph coordinate system.
Coordinate system used to generate a kymograph tracking kinetochore motion measured along the line p_1 to p_2 which passes through the kinetochore sisters s_1 and s_2. The line is maintained at constant distance *d* from the metaphase plate and with constant length *l* to allow visualisation of kinetochore oscillations. See Methods for details.

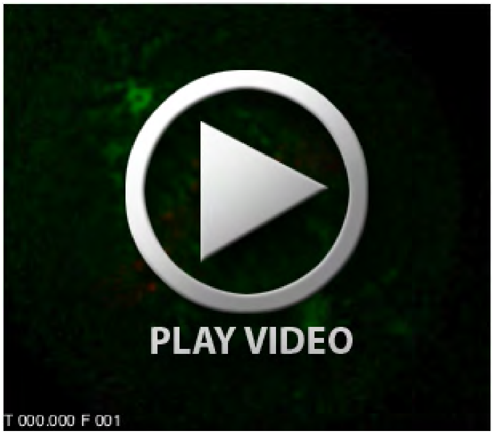

Movie 1. Cell expressing mCherry-CENP-A (red) and EB3-eGFP (green), corresponding to Figure 1.
Frame size is 17.2 x 19.5 μm and frame time-step is 2 s.

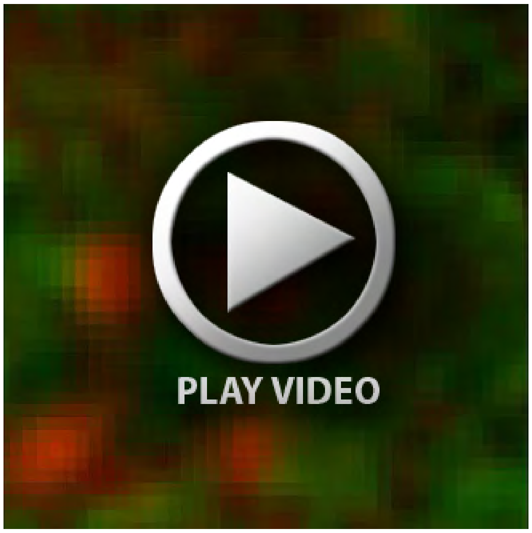

Movie 2. Kinetochore labelled with mCherry-CENP-A (red) with EB3-eGFP bursts (green), corresponding to Figure 1.
Frame size is 3.5 x 3.5 μm and frame time-step is 2s. Movie is centred on kinetochore using tracking data.

	Wild-type	*Control* siRNA	*MCAK* siRNA	*KIF18A* siRNA
Cells	147	170	92	105
Kinetochore (KT) tracks after filtering	850	850	350	469
Tracks with anti-poleward (AP) bursts[1]	197	198	57	94
Tracks with poleward (P) bursts	66	80	35	28
Tracks with both AP and P bursts	17	11	4	5
Runs	1923	1847	698	977
Runs with AP bursts	213	219	59	99
Runs with P bursts	66	80	35	28
AP bursts	457	433	108	202
P bursts	160	177	67	70
AP bursts in runs	248	242	66	114
P bursts in runs	71	88	37	31
Bursts occurring in directionless segments	366	412	161	200

Table S1. Kinetochore tracking statistics. Runs and bursts were located as described in Methods.

[1]Tracks with AP or P bursts are not mutually exclusive since a track may have bursts in both P and AP runs.

Table S2.

Download Table S2

Dataset	Mean of maximum mask intensity	Estimated number of polymerising MTs
P KTs (all tracks)	1.44±0.50	9±1
AP KTs (all tracks)	1.60±0.63	10±2
AP KTs (tracks with AP burst)	1.87±0.70	12±3
P KTs (tracks with AP burst)	1.45±0.53	9±1
AP KTs (tracks without bursts)	1.49±0.55	9±2
P KTs (tracks without bursts)	1.42±0.48	9±1
AP KT burst frames	2.68±0.76	19±3
P KT burst frames	2.46±0.69	17±3

Table S3. Estimation of number of polymerising microtubules. Experiments with recombinant proteins have shown that there is a linear relationship between EB protein binding at the MT tip and the growth speed. In our data, we found the speed of KTs to be, on average, approximately ten times slower than astral MTs. Consequently, we can estimate the number of polymerising MTs by comparing EB3 intensity to that of single astral MTs and scaling for the difference in speed (faster MTs have proportionally higher levels of EB3 accumulation (Bieling et al., 2007; Straube, 2011); Anne Straube, personal communication). Contamination from spindle background in the KT mask can be compensated for by the mean KT mask intensity (Fig. S1; see also main text). The number of polymerising MTs was calculated as (astral speed / KT speed) * (mean of maximum KT mask intensity - mean KT mask intensity) / mean of maximum astral intensity.

	**control siRNA**	***MCAK* siRNA**	***KIF18A* siRNA**	**control for alternate siRNAs**	***MCAK* alternate siRNA**	***KIF18A* alternate siRNA**
Cells	170	92	105	99	10	68
Kinetochore (KT) tracks after filtering	850	350	469	1099	61	328
Runs	1847	698	977	1401	159	667
Ratio of AP to P bursts (as in Fig. 6B).	2.45	1.61	2.89	2.1	1.2	2.4
Bias to AP (as in Fig. 6C)	12.0%	3.9%	17.1%	11.6%	4.3%	15.4%
Intrinsic bias to AP (as in Fig. 6D)	6.5%	0.9%	12.6%	7.0%	2.8%	11.1%

Table S4. Confirmation of MCAK depletion and KIF18A depletion phenotypes by independent siRNA. The alternate siRNAs are detailed in the Methods section of the main text.

References

Bieling, P., Laan, L., Schek, H., Munteanu, E. L., Sandblad, L., Dogterom, M., Brunner, D. and Surrey, T. (2007). Reconstitution of a microtubule plus-end tracking system in vitro. *Nature* **450**, 1100-1105.

Straube, A. (2011). How to Measure Microtubule Dynamics? In *Microtubule Dynamics*, vol. 777 (ed. A. Straube), pp. 1-14: Humana Press.
